# Biocompatibility and Drug Release Properties of Carboxymethyl Cellulose Hydrogel for Carboplatin Delivery

**DOI:** 10.3390/gels12010005

**Published:** 2025-12-20

**Authors:** Hiroyuki Kono, Shion Kinjyo, Ryou Uyama, Sayaka Fujita, Yuko Murayama, Shinya Ikematsu

**Affiliations:** 1 Division of Applied chemistry and Biochemistry, National Institute of Technology, Tomakomai College, Nishikioka 443, Tomakomai 059-1275, Japan; 2 Department of Bioresources Engineering, National Institute of Technology, Okinawa College, Henoko 905, Nago 905-2192, Japan

**Keywords:** biocompatibility, carboplatin, carboxymethylcellulose, cell culture, colorectal cancer cells, controlled release, drug delivery system, hydrogel, in vivo study, safety evaluation

## Abstract

Carboxymethylcellulose (CMC) is a biocompatible and biodegradable polysaccharide suitable for biomedical applications. Herein, an epichlorohydrin (ECH)-crosslinked CMC hydrogel (CMCG) was developed as a carrier for sustained drug release. Ether-type crosslinking between the hydroxyl groups of CMC and ECH yielded a transparent, highly water-absorbent gel. Structural analyses employing Fourier-transform infrared and solid-state ^13^C nuclear magnetic resonance spectroscopies confirmed successful crosslinking, and the hydrogel exhibited pH-dependent swelling. Carboplatin (CBP), a platinum-based anticancer drug, was incorporated into CMCG to prepare CBP-CMCG. In phosphate-buffered saline (pH 7.4), approximately 70% of CBP was released within 12 h, followed by a plateau phase, indicating diffusion-controlled release. Cytocompatibility assays using WI-38 normal human fibroblasts demonstrated that CMCG was non-cytotoxic, whereas free CBP induced significant cell death. In colorectal cancer HT-29 cells, CBP-CMCG exhibited gradual cytotoxicity, resulting in >80% nonviable cells after 24 h, indicating a sustained antitumor effect compared with free CBP. These results demonstrate that the newly developed ECH-crosslinked CMC hydrogel is a safe and effective platform for controlled drug delivery, enabling sustained release and prolonged therapeutic activity of CBP.

## 1. Introduction

Hydrogels derived from natural polysaccharides and their derivatives have attracted significant attention in recent decades owing to their inherent safety, biocompatibility, and biodegradability [1,2]. These materials are particularly promising for biomedical applications, where long-term contact with living tissues requires both minimal toxicity and sufficient functional performance. In drug delivery systems, hydrogels offer distinct advantages, such as the ability to encapsulate therapeutic agents within their three-dimensional networks and controlled release in response to environmental stimuli. For these reasons, polysaccharide-based hydrogels have been investigated for a broad spectrum of medical uses, ranging from wound dressings and tissue scaffolds to carriers for small molecules and biologics [3,4,5].

Hydrogels are generally classified into two categories: chemical and physical gels [6]. Chemical hydrogels are fabricated by covalently crosslinking polymer chains via reactive crosslinkers, thereby forming stable, permanent networks. This covalent architecture confers high structural stability, resistance to dissolution, and superior mechanical properties [7]. However, these advantages often compromise biocompatibility, as many crosslinkers are highly reactive and may generate cytotoxic residues within the material [8]. Physical hydrogels are formed via reversible noncovalent interactions such as hydrogen bonding, ionic interactions, and hydrophobic effects. Although they offer improved biocompatibility, they exhibit weaker mechanical stability and are prone to rapid disintegration under physiological conditions. This trade-off between stability and safety foments the development of hydrogel systems that can reconcile these conflicting requirements [9,10].

Among polysaccharide-based materials, CMC has emerged as an attractive hydrogel precursor due to its water solubility, chemical versatility, and excellent safety profile [11,12]. CMC-based hydrogels have been widely investigated for drug encapsulation and controlled release, with numerous studies reporting their physicochemical properties and in vitro release kinetics [13,14,15]. However, despite this extensive research, biological evaluations of CMC hydrogels remain limited. Most studies have focused on passive drug release behaviors rather than their cytocompatibility, cellular responses, or therapeutic performance in biological systems [12,16,17,18].

This gap is particularly evident for platinum-based chemotherapeutics such as CBP, which require controlled release and biocompatible carriers to minimize systemic toxicity. While CMC hydrogels represent a promising platform for such drugs, their biosafety and antitumor efficacy have not been sufficiently examined at the cellular level.

In the present study, we synthesized a chemically crosslinked hydrogel by crosslinking CMC with ECH, ensuring structural robustness [11,12]. CBP, a platinum-based chemotherapeutic agent, was incorporated into the CMCG network. Unlike previous studies that have primarily restricted their focus to in vitro release kinetics, our work emphasizes biological performance by evaluating both the biosafety of the hydrogel and the antitumor efficacy of the CBP-loaded system at the cellular level. Therefore, our approach distinguishes from previous studies by moving beyond physicochemical analysis to demonstrate therapeutic functionality in cancer cell models [18]. Furthermore, demonstrating that CMCG maintains biocompatibility while enabling sustained CBP release and effective antitumor activity represents a significant advance in the field. Such a system holds the potential to expand the biomedical applications of chemically crosslinked hydrogels, bridging the gap between controlled release studies and clinically relevant therapeutic outcomes.

## 2. Results and Discussion

### 2.1. Preparation and Characterization of CMCG

#### 2.1.1. Preparation and Structural Characterization

**Figure 1:**
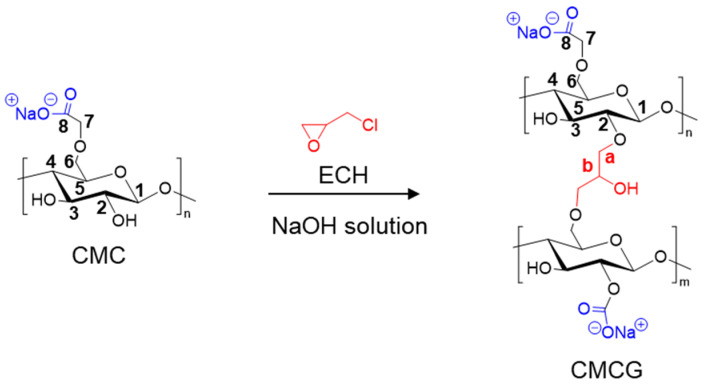
CMCG synthesis via crosslinking of CMC with ECH. ECH reacts with the unsubstituted hydroxyl groups of CMC, leading to the formation of ether-type crosslinks.

CMC-based hydrogels are typically synthesized by reacting CMC with bifunctional crosslinkers such as ethylene glycol diglycidyl ether as well as ECH, employing the unsubstituted hydroxyl groups of CMC that serve as crosslinking sites to form intermolecular networks [11,12,19,20]. In this study, CMCG was prepared via dropwise addition of ECH to a CMC solution in aqueous sodium hydroxide (NaOH), following a previously reported method [11,21] (Figure 1). Upon ECH addition, the viscosity of the transparent CMC solution immediately increased; subsequent heating at 60 °C induced a gradual sol–gel transition. After reacting for 3 h, a transparent gel was obtained, crushed, and thoroughly washed with a water/ethanol mixture. The product was then dialyzed against running water for 3 days to completely remove unreacted ECH, residual NaOH, and other impurities. After vacuum drying, the material was passed through a 40-mesh sieve to yield a white, granular CMCG. A yield of 8.9 g of CMCG was achieved from 10 g of CMC.

**Figure 2:**
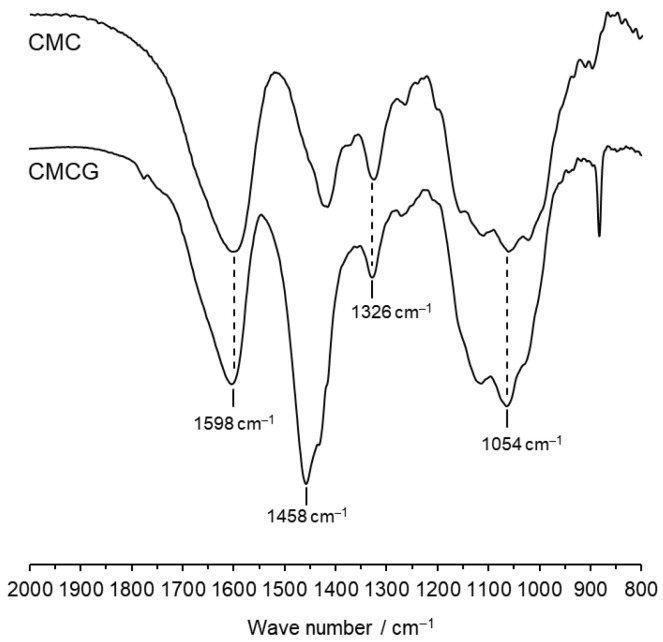
FTIR spectra of CMCG and CMC. These spectra were normalized by the peak intensity of the carboxylate bands at 1598 cm^−1^.

Figure 2 shows the Fourier-transform infrared (FTIR) spectra of CMCG and the starting CMC. Spectra were normalized to the absorption band of the carboxylate group at 1598 cm^−1^, which does not participate in the crosslinking reaction. Characteristic bands at 1326 and 1054 cm^−1^ corresponded to C–H bending and asymmetric stretching of the C–O–C group, respectively [21]. In the CMCG spectrum, the absorption intensity at 1054 cm^−1^ was increased relative to CMC, and a new absorption band appeared at 1458 cm^−1^, corresponding to CH_2_ bending vibrations [19]. These results confirm that ECH reacted with the hydroxyl groups of CMC to form crosslinked structures.

**Figure 3:**
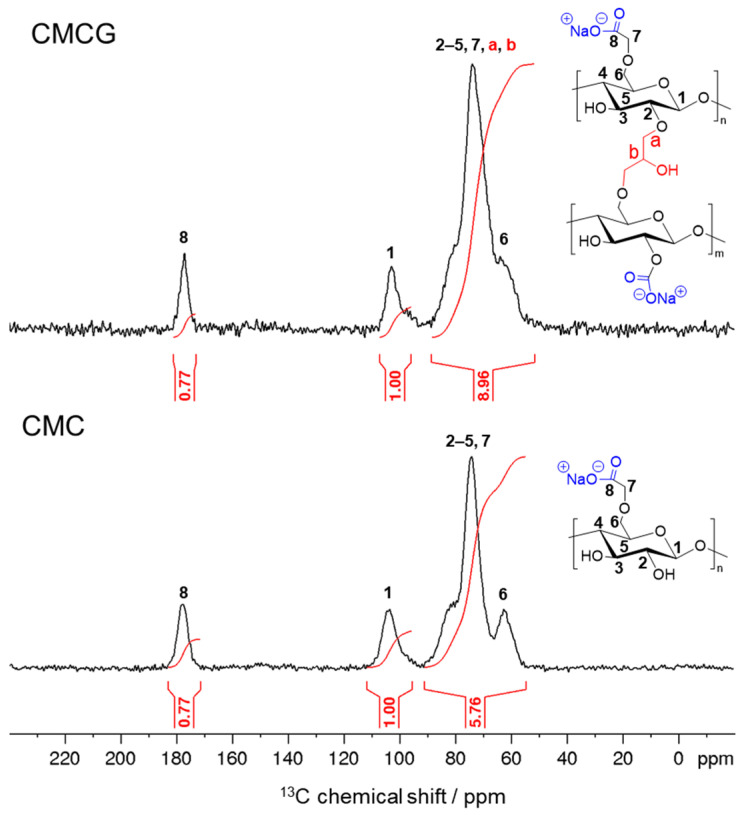
Quantitative solid-state ^13^C NMR spectra of CMCG and CMC. ^13^C resonance assignment and integrals are shown in these spectra.

Quantitative solid-state ^13^C nuclear magnetic resonance (NMR) spectroscopy was performed to determine the amount of ECH molecules crosslinked per anhydroglucose unit (AGU) of CMC in the resulting CMCG (Figure 3). In the starting CMC spectrum, ^13^C resonances were observed at 178, 103, and 63 ppm, corresponding to the carboxyl carbon, C1, and C6, respectively; the other carbons (C2–C5 and methine carbons) were associated with broad overlapping resonances in the 92–67 ppm region [21]. From the integral ratio of the carboxyl carbon to C1, the degree of substitution (DS) of the CMC was calculated as 0.77. The 92–55 ppm region contained the five cellulose carbons (C2–C6) and the methine carbons of the carboxymethyl groups. Given that the integral of the methine carbons is equivalent to that of the carboxyl carbons (0.77), the theoretical integral value for the 92–55 ppm region is 5.77, consistent with the measured value of 5.76. This confirms the quantitative reliability of the solid-state ^13^C NMR method.

In the CMCG spectrum the relative intensity of the carboxyl carbon resonance (0.77) remained constant, confirming that the carboxyl groups were not involved in the crosslinking reaction for the preparation of CMCG. The integral value of the 92–55 ppm region increased from 5.76 to 8.96, attributable to ECH crosslinking with CMC. Based on these results, the number of ECH molecules reacted per AGU of CMC (the crosslinking ratio) was calculated to be 1.07 using the following Equation (1):

(crosslinking ratio) = (8.96 − 5.76)/3 = 1.07(1)

where the denominator (3) represents the number of carbon atoms in ECH. During crosslinking, the cleavage of the ECH epoxy ring generates a hydroxyl group (Figure 1), which serves as a new reactive site for further reactions, resulting in a high crosslinking density.

#### 2.1.2. Water-Absorbency

**Figure 4:**
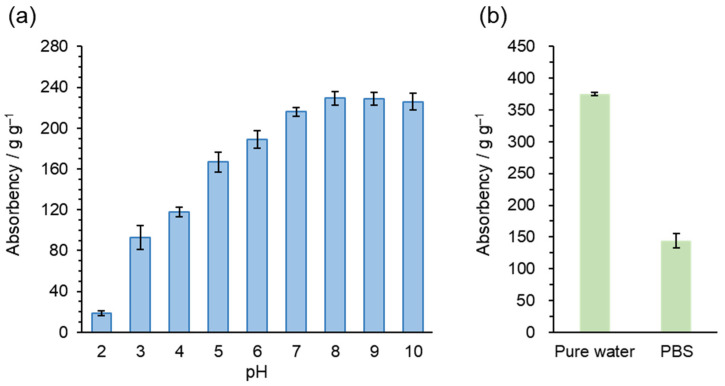
Saturated water absorption of CMCG: (**a**) saturated absorption in 20 mM buffer solutions (pH 2–10); (**b**) saturated absorption in pure water and PBS solution.

The saturated water absorption of CMCG is presented in Figure 4. The water uptake of CMCG under different pH buffer conditions (20 mM salt concentration) substantially increased as buffer pH increased (Figure 4a), a behavior attributed to the ionization of carboxyl groups in the polymeric structure [21,22,23]. In neutral and alkaline environments, the predominant species are anionic carboxyl groups; electrostatic repulsion among these groups causes hydrogel swelling. At acidic pH, the carboxyl groups are protonated and are electrostatically neutralized, reducing or eliminating electrostatic repulsion and preventing swelling, thereby resulting in relatively low water uptake. Figure 4b shows the saturated water absorption of CMCG in pure water and PBS, which were 375 and 140 g g^−1^, respectively. Water absorption in ionic gels such as CMCG depends on the osmotic pressure differential generated by the difference in mobile cation concentrations between the gel and saline solution. In PBS, the higher external ionic strength reduces this osmotic pressure difference, thereby decreasing water uptake compared with pure water [21,23].

**Figure 5:**
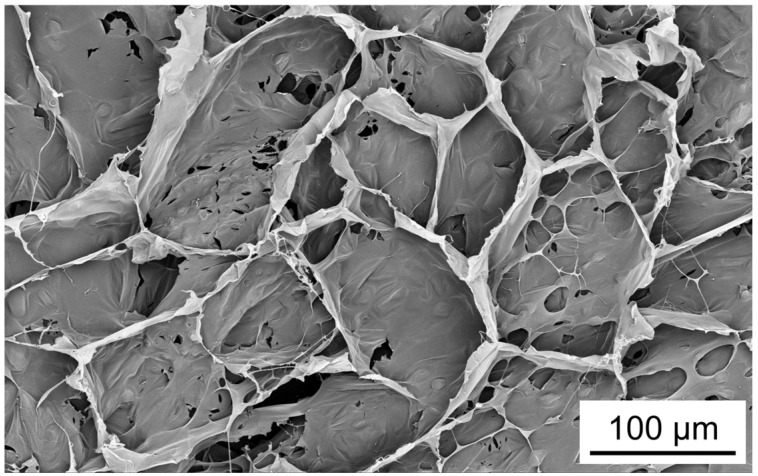
SEM image of cross-sectional surface of PBS-saturated CMCG.

SEM imaging cross-section of the swollen hydrogel revealed a porous morphology characteristic of swollen hydrogels (Figure 5). This indicates that a three-dimensional network was formed via the crosslinking of CMC with ECH, effectively retaining within the structure [21,22]. On this basis, if CMCG is swollen in drug-containing aqueous solutions, this three-dimensional network serves as a molecular diffusion barrier, potentially facilitating sustained drug release.

### 2.2. Preparation of CBP-CMCG and CBP Release In Vitro

Polysaccharide-based hydrogels are highly suitable materials for sustained drug release and delivery [24,25]. Performance evaluation demands comprehensive assessment of both release kinetics and the safety profile of the hydrogel, as these factors directly impact its therapeutic efficacy and side effects [18,25]. To investigate CMCG as a drug delivery matrix, CBP was selected as a model drug, incorporating it into CMCG to evaluate in vitro release characteristics. CBP inhibits DNA synthesis during cancer cell division, thereby suppressing cell proliferation and inducing apoptosis [26,27]. For this reason, it is widely used in the treatment of head and neck cancer, small cell lung cancer, testicular tumors, ovarian cancer, cervical cancer, malignant lymphoma, non-small cell lung cancer, breast cancer, and as part of chemotherapy regimens for pediatric malignant solid tumors [26,27,28]. Furthermore, CBP is water-soluble, which facilitates efficient incorporation into the CMCG hydrogel network [18,24].

**Figure 6:**
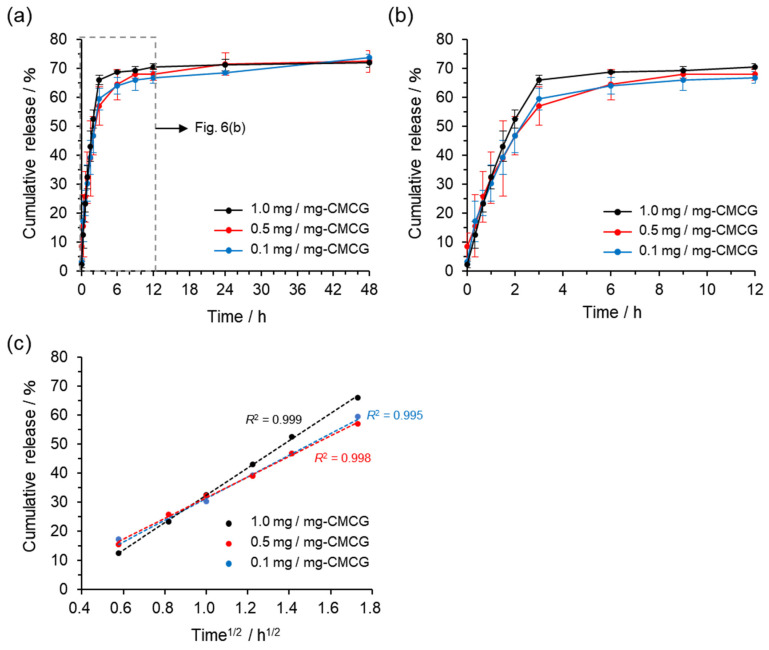
CBP release behavior of CBP-CMCG in PBS at 37 °C. (**a**) CBP release up to 48 h, (**b**) initial CBP release (up to 12 h), and (**c**) initial CBP release as a function of the square root of release time.

CMCG was swollen in a CBP aqueous solution, homogenized, rapidly frozen in liquid nitrogen, and subsequently lyophilized to yield CBP-CMCG with drug loadings of 0.1, 0.5, and 1.0 mg/mg-CMCG. Release profiles were obtained by immersing CBP-CMCG samples in PBS (pH 7.4) and monitoring the cumulative release over time (Figure 6a,b). Because the lyophilized CMCG was crushed and sieved through a 40-mesh filter prior to release testing, the hydrogel particles used in the experiments were smaller than approximately 400 µm and exhibited irregular, non-uniform shapes. Although the size and morphology were not strictly controlled, the diffusion-controlled release observed in this study reflects the average behavior of these heterogeneous particles. The mesh size of CMCG is considerably larger than the molecular dimensions of CBP. In addition, both the hydrogel matrix and CBP are highly hydrophilic. Because of these factors, variations in particle shape and size are expected to exert only a minor influence on the intrinsic molecular diffusion process. This is consistent with the similar release profiles previously obtained across various CBP loading levels [29,30].

CBP-CMCG gradually released CBP regardless of the loading amount. Approximately 70% of the encapsulated drug was released within the first 12 h [18,29]. The cumulative release after 24 h was nearly identical to 12 h values, indicating that equilibrium was reached within 12 h [18]. Furthermore, no burst release (a common feature in conventional carriers) was detected during the test, suggesting that CBP was uniformly distributed within the CMCG matrix [29,30].

The initial release of CBP from CMCG was proportional to the square root of the release time (Figure 6c). This relationship indicates that the mesh size formed by the CMCG network is substantially larger than the molecular size of CBP. It also suggests that the mesh structure of CMCG functions as a diffusion barrier for CBP [31,32]. The plateau following the release of 70% of CBP after 12 h suggests that the remaining encapsulated drug was bound to the hydrogel matrix. Given the divalent platinum center in CBP, we propose that electrostatic interactions between CBP and the anionic carboxylate groups of CMC contributed to this retention [33]. This hypothesis aligns with studies demonstrating that CMC hydrogels with high carboxyl group density exhibit large mesh sizes and strong ion-polyelectrolyte interactions [34]. Quantitative analyses such as isothermal titration calorimetry, dialysis binding assays, and spectroscopy will be necessary to verify this interaction.

These results suggest that CBP is retained within the CMCG network and released steadily over time, primarily via molecular diffusion. During this process, electrostatic binding with carboxymethyl groups of CMCG likely immobilized a fraction (~30%) of the drug. By varying the crosslinking ratio of CMCG, the network voids (mesh size) can be tuned, enabling the control of the CBP release rate [34].

Importantly, the release study was conducted under sink conditions. CBP has a solubility of approximately 10–14 mg/mL in aqueous media [35], which is substantially higher than the CBP concentrations used in the release medium. Therefore, the CBP concentration did not approach saturation during the experiments, confirming that the obtained release profiles reflect intrinsic diffusion behavior and drug–matrix interactions rather than solubility-limited effects.

Although CBP is widely used in long-term chemotherapy, herein, the drug release was evaluated only up to 48 h. CBP is highly hydrophilic and typically exhibits relatively rapid diffusion from hydrophilic polymer matrices, including CMC-based hydrogels. Previous reports on CMC hydrogels have also demonstrated release durations within 24–72 h [36,37], which is consistent with our observations. Nevertheless, longer-term release evaluation (days to weeks) is necessary to fully assess the suitability of this system for sustained chemotherapy, which will be investigated in future work.

### 2.3. Evaluation of the Cytocompatibility of CMCG

The biocompatibility of CMCG was evaluated via cytocompability assays with the normal WI-38 cell line, a normal fibroblast cell line derived from the lung tissue of a female embryo three months post-fertilization [36,37]. After subculture, WI-38 cells were incubated for 24 h under standard conditions. CMCG was then added to the culture medium until a concentration of 10 mg/mL was achieved, and the WI-38 cells were cultured for an additional 24 h. For comparison, cells treated with CBP (3.0 mg/mL) served as a positive control for cytotoxicity.

**Figure 7:**
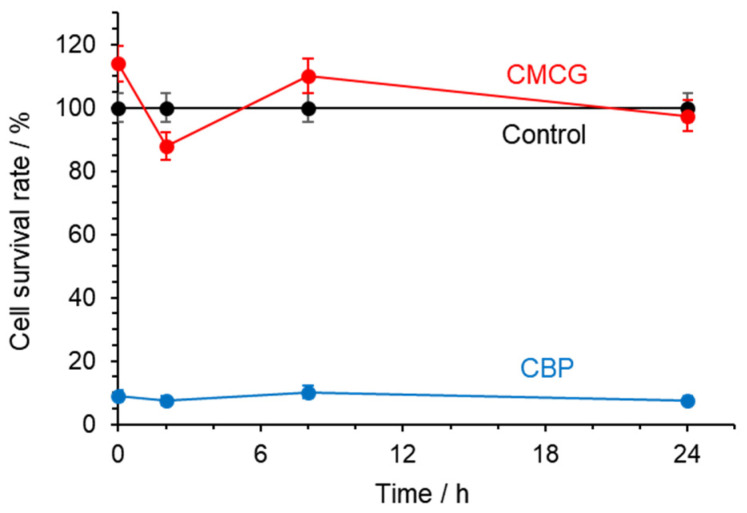
Cell survival rate of the WI-38 cell line in the presence of CMCG and CBP.

WI-38 cells cultured in the presence of CMCG showed minimal fluctuations in cell number (Figure 7). No significant changes in cell proliferation were observed compared with the untreated control. These observations are consistent with previous reports demonstrating the negligible cytotoxicity of CMC-based hydrogels in fibroblast cell lines [29]. In comparison, exposure to CBP resulted in a substantial decrease in cell number immediately after addition, with only about 10% of cells surviving, which agrees with earlier findings on CBP cytotoxicity [38,39].

**Figure 8:**
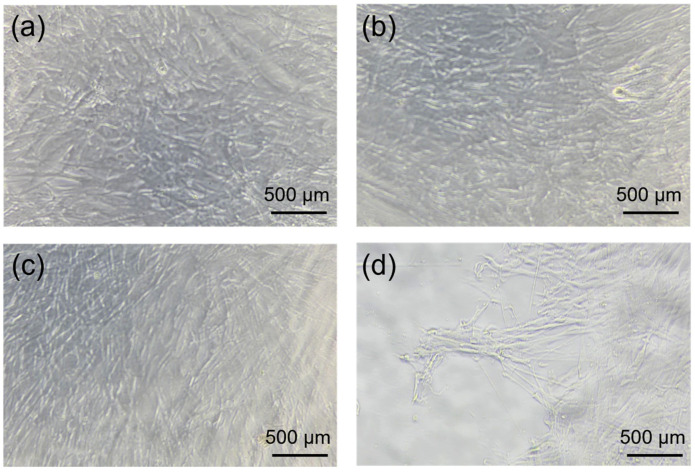
Optical micrographs of WI-38 cell line after: (**a**) 0 h, (**b**) 24 h, (**c**) 24 h in the presence of CMCG, and (**d**) after 24 h in the presence of CBP.

Microscopic observation of WI-38 cells before and after culture confirmed that CMCG did not inhibit cell growth (Figure 8). The WI-38 cells maintained nearly identical morphology and density at the start of culture and after 24 h (Figure 8a,b). WI-38 cells cultured in the presence of CMCG also retained normal morphology and density (Figure 8c). The CBP-treated group exhibited extensive cell death, with nearly no viable cells observed within the microscopic field of view (Figure 8d), which aligns with established findings that CBP induces significant cytotoxicity in normal fibroblast-like cell [40]. These results based on cell culture demonstrate that CMCG does not exhibit cytotoxicity toward normal cells, suggesting that it is a highly biocompatible and safe material.

In the cytocompatibility evaluation using normal fibroblasts, we focused on assessing the intrinsic cytotoxicity of CMCG. CBP-CMCG was not tested on normal cells because the CBP doses used in the cancer-cell cytotoxicity assays would be excessively toxic to normal fibroblasts, making the results difficult to interpret and less meaningful. Therefore, the primary objective of this assay was to confirm that the hydrogel scaffold itself did not induce cytotoxicity. The evaluation of the effects of CBP-CMCG on normal cells will be conducted in future studies.

Moreover, the biodegradation behavior of CMCG was not examined in this study. Although CMC is generally regarded as an enzymatically degradable and biocompatible polysaccharide, the in vivo degradation behavior of CMCG has not yet been fully characterized. In particular, it remains unclear how these hydrogels degrade under physiological conditions and whether any potentially toxic degradation byproducts may be generated. A comprehensive assessment of long-term degradation kinetics and identification of possible degradation products will therefore be essential for future biomedical applications.

### 2.4. Sustained Release Effect of CBP-CMCG in Colorectal Cancer Cells

After confirming the cytocompatibility of CMCG, the functionality of CBP-CMCG as a sustained-release formulation was evaluated in vitro. CBP-CMCG samples were prepared with a CBP: CMCG ratio of 1.0 mg:1.0 mg and used for cell culture experiments. Human colorectal cancer cells (HT-29 cell line) were cultured and divided into three groups: cells treated with CMCG alone (negative control), cells treated with CBP-CMCG, and cells treated with CBP alone (positive control).

**Figure 9:**
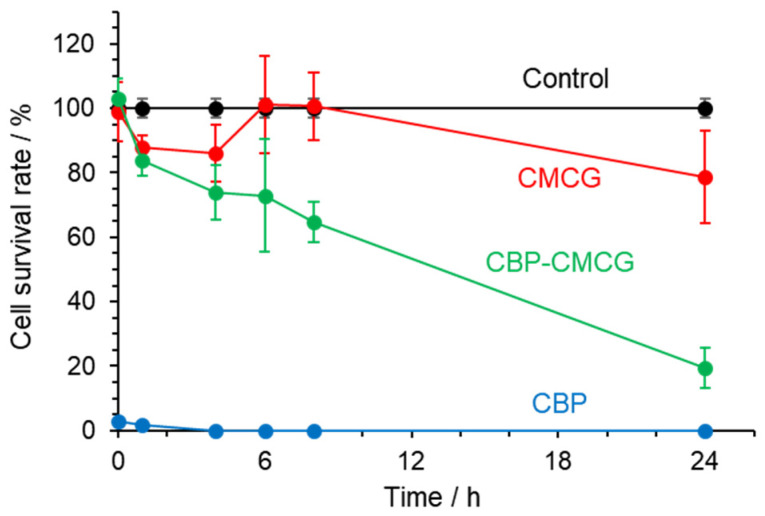
Cell survival rate of HT-29 cells in the presence of CMCG, CBP, and CBP-CMCG.

As shown in Figure 9, CMCG alone exhibited no apparent cytotoxicity toward HT-29 cells [41]. By contrast, CBP alone showed strong anticancer activity, with nearly all cells dying within 1 h after treatment [42]. Meanwhile, in the presence of CBP-CMCG, the cell survival rate gradually decreased over time. These results indicate that CBP was gradually released from the CMCG matrix and exerted a sustained anticancer effect with time [40], confirming its effectiveness as a CBP carrier.

**Figure 10:**
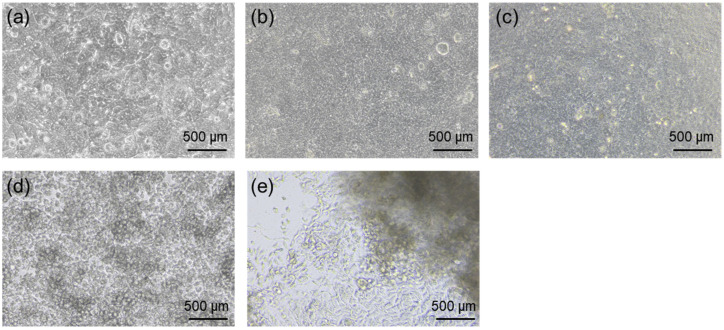
Optical micrographs of HT-29 cells: (**a**) at 0 h, (**b**) after 24 h, (**c**) after 24 h in the presence of CMCG, (**d**) after 24 h in the presence of CBP-CMCG, and (**e**) after 24 h in the presence of CBP.

Microscopic observation of HT-29 cells supported that CMCG functions as a sustained-release matrix (Figure 10). HT-29 cells cultured under normal conditions at the start of culture (Figure 10a) and after 24 h (Figure 10b) exhibited typical adherent morphology with multilayered proliferation [41]. A similar morphology was observed in the presence of CMCG (Figure 10c), indicating that CMCG exerted negligible inhibition on HT-29 cells, being consistent with WI-38 cells [42]. By contrast, cells treated with CBP alone showed near-total cell death (Figure 10e) [2]. Under CBP-CMCG conditions, although cell density decreased, continued proliferation was confirmed (Figure 10d). This confirms that CMC-based hydrogels enable the sustained release of incorporated drugs, resulting in gradual cytotoxic effects on cancer cells [41].

These findings demonstrate that CMCG is not only biocompatible and non-toxic, but also a viable reservoir for anticancer drugs, enabling continuous release. Therefore, via precise molecular design that includes controlling molecular weight of the starting material of CMC and tuning the crosslinking ratio, CMCG is expected to function as an effective drug carrier capable of delivering controlled therapeutic effects over an extended period.

## 3. Conclusions

We developed and evaluated CMCG as a biocompatible platform for sustained drug delivery. This hydrogel exhibited high water absorption, stable network formation, and pH-dependent swelling behavior. Cytocompability assays using normal human WI-38 fibroblasts confirmed that CMCG is non-toxic, maintaining normal cell morphology and proliferation. Furthermore, CBP-CMCG exhibited sustained anticancer activity against colorectal cancer HT-29 cells through gradual drug release, in sharp contrast to the rapid cytotoxicity observed with free CBP.

These findings indicate that the newly developed CMCG possesses both the safety and functionality required for biomedical applications. Taken together, the results demonstrate that this system provides a promising platform for controlled and sustained drug delivery, as well as for tissue engineering research. Further studies are warranted to clarify the in vivo properties and therapeutic efficacy of both CBP-CMCG and CMCG through mechanistic evaluations such as apoptosis assays, comparisons with other hydrogel systems, and biological assessments using additional cell lines.

## 4. Materials and Methods

### 4.1. Materials and Cells

CMC (viscosity-average molecular weight = 1.2 × 10^5^) was purchased from CP Kelco Co., Ltd., Tokyo, Japan. ECH was obtained from Tokyo Chemical Industry Co., Ltd., Tokyo, Japan. CBP of pharmaceutical primary standard grade was purchased from Sigma-Aldrich Co. LLC, St. Louis, MO, USA.

Human normal fibroblast cell line WI-38 human colorectal adenocarcinoma cell line HT-29 were obtained from the RIKEN BioResource Research Center, Japan. Minimum essential Medium (MEM) and Gibco™ penicillin–streptomycin (PS, 10,000 U/mL) were purchased from Thermo Fisher Scientific Inc., Waltham, MA, USA. HyClone™ USDA-import-tested fetal bovine serum (FBS) was obtained from Global Life Sciences Technologies Japan K.K., Tokyo, Japan. The prime WST-1 cell proliferation assay system was purchased from Takara Bio Inc., Tokyo, Japan. Dulbecco’s modified eagle’s medium (DMEM; high glucose, containing sodium bicarbonate and L-glutamine but without sodium pyruvate) was obtained from Merck KGaA, Darmstadt, Germany.

All other chemicals were of reagent grade, purchased from FUJIFILM Wako Pure Chemical Co., Tokyo, Japan, and used without further modification.

### 4.2. Preparation of CMCG

CMC (25.0 g, 110 mmol for AGU) was completely dissolved in 100 mL of 1.5 mol L^−1^ aqueous NaOH solution, and ECH (0.77 g, 4.4 mmol) was subsequently added to the solution with stirring at 300 rpm using a Teflon impeller at 25 °C. After 10 min, the crosslinking reaction was performed at 60 °C while stirring at 300 rpm for 3 h. The reaction mixture was washed twice with a solution of deionized water and ethanol (1:1), and the resulting mixture was dialyzed against distilled water using a dialysis membrane (MW 12,000–13,000 cut). The obtained hydrogel was freeze-dried and subsequently screened through a 40-mesh sieve to obtain the white granular CMCG.

### 4.3. Characterization of CMCG

#### 4.3.1. Structural Characterization

FTIR spectra were recorded using an ALPHA II FTIR spectrometer (Bruker Optics GmbH & Co., KG, Bremen, Germany) at 295 K. After grinding the sample into a powder and mixing it with KBr powder, the mixture was compressed into a transparent disk and scanned from 4000 to 500 cm^−1^, with an average of 16 scans and a resolution of 1 cm^−1^.

Solid-state ^13^C NMR experiments were performed at 295 K on an AVIII500 spectrometer equipped with a double-resonance 4 mm magic angle spinning (MAS) probe (Bruker BioSpin GmbH, Ettlingen, Germany). Each sample (approximately 50 mg) was packed in the Bruker 4 mm ZrO_2_ rotor, and the MAS frequency of the sample rotor was set to 10 kHz. Solid-state ^13^C NMR spectra were recorded using the dipolar-decoupled/MAS method with a flip angle for ^13^C excitation pulse of 30°, a repetition time of 30 s, and 4096 scans for quantitative evaluation. The ^13^C chemical shifts in the solid-state ^13^C NMR spectra were referenced against the carbonyl carbon resonance of D-glycine as an external reference (176.03 ppm).

SEM images of the swollen CMCG cross-sectional structure were obtained using the following sample preparation method: After swelling in deionized water at 25 °C for 48 h, the CMCG was carefully cut with a razor blade, frozen at −70 °C, and freeze-dried under vacuum until the water sublimated. SEM images of the dry CMCG cross-sectional structure were obtained by cutting with a razor blade and observing directly. These samples were fixed on a specimen stub using carbon tape and sputter-coated with platinum prior to observation. SEM images were acquired using a Phenom Pro G6 scanning electron microscope (Thermo Fisher Scientific Inc., Waltham, MA, USA) with a potential of 1 kV.

#### 4.3.2. Determination of Water-Absorbency

The water absorbency of CMCG was evaluated using the tea bag method [43] in accordance with the Japan Industrial Standard (JIS K7223 [43]. CMCG (100 mg) was placed in a tea bag (50 × 100 mm) made of a 225-mesh nylon sheet, and the bag was immersed in pure water at 298 K. After a predetermined period, the tea bag was removed from the solution and allowed to drain for 10 min to remove excess surface water. The weight of the tea bag containing the swollen CMCG (W_h_) was then measured, and the water absorbency was calculated using Equation (2):

Water absorbency = (W_h_ − W_b_ − W_d_)/W_d_, (2)

where W_b_ is the weight of the empty tea bag after water treatment and Wd is the weight of the dried CMCG. Each absorbency measurement was performed five times for each solution. For the water absorption tests, pure water, 20 mM citric acid–sodium citrate buffer solutions (pH 2–5), 20 mM phosphate buffer solutions (pH 6–8), 20 mM boric acid–sodium borate buffer solutions (pH 9–10), and a PBS buffer solution (pH 7.4) were used. The water absorbency values are reported as the mean of five measurements (*n* = 5), and the standard deviations are presented as error bars.

### 4.4. Preparation and Characterization of CBP-CMCG

#### 4.4.1. Preparation of CBP-CMCG

CMCG (100 mg) was added to 10 mL of an aqueous CBP solution and allowed to swell for 2 h at 4 °C. The swollen hydrogel was then homogenized using a T25 digital Ultra-Turrax disperser (IKA-Werke GmbH, Staufen, Germany) at 15,000 rpm for 3 min and lyophilized to obtain CBP-CMCG.

CBP-CMCG samples were prepared with CBP solutions at concentrations of 1, 5, and 10 wt%, corresponding to CBP loadings of 0.1, 0.5, and 1.0 mg CBP per mg CMCG, respectively.

#### 4.4.2. CBP Release from CBP-CMCG

CBP-CMCG (5 mg) was placed in a microtube containing 1.5 mL of PBS and incubated at 37 °C. At predetermined time intervals, 50 μL of the supernatant was withdrawn and replaced with an equal volume of fresh PBS to maintain a constant total volume. The CBP concentration in each supernatant was measured via absorbance at 236 nm using a Spark microplate reader (Tecan Trading AG, Männedorf, Switzerland) and quantified from a standard calibration curve of CBP. All release experiments were performed in triplicate. The amounts of CBP released were reported as the mean of three independent experiments (*n* = 3), and the standard deviations were represented as error bars.

### 4.5. Safety Evaluation of CMCG by Cell Viability Assay

WI-38 cells were cultured in MEM supplemented with 10% FBS and 1% PS. Subcultured WI-38 cells were seeded into 96-well plates at a density of 1 × 10^4^ cells/well and incubated for 24 h at 37 °C in a 5% CO_2_ incubator. WI-38 cells used for the WST-1 assay were subcultured once prior to seeding. After incubation, the culture medium was completely removed using an aspirator. CMCG (1.0 mg) swollen in 110 µL of DMEM containing 10% FBS was then added to each well. The positive control was 110 µL of DMEM containing 10% FBS and 1.0 mg of CBP, and the negative control was 110 µL of DMEM containing 10% FBS. After each sample addition, 10 µL of WST-1 reagent was added at 0, 2, 4, 8, and 24 h. After 2 h of incubation, absorbance at 440 nm (reference wavelength 660 nm) was measured using a SpectraMax Mini multimode microplate reader (Molecular Devices, LLC., San Jose, CA, USA). Cell viability was calculated using the following Equation (3):

Cell viability (%) = [(Abs_sample_ − Abs_medium_)/(Abs_control_ − Abs_blank_)] × 100 (3)

where Abs_sample_, Abs_medium_, Abs_control_, and Abs_blank_ are the absorbances of the sample, medium, control culture (cells only), and blank (medium only), respectively. Cell viability was determined as the mean of three independent experiments (*n* = 3), with standard deviations represented as error bars.

### 4.6. Evaluation of the Sustained Release of CBP from CBP-CMCG in Cancer Cells

#### 4.6.1. Preparation of Cancer Cell Line

Cancer cell line: HT-29 cells (human colorectal adenocarcinoma) were obtained from the RIKEN Bioresource Research Center, Japan. For cell culture, DMEM supplemented with 10% fetal bovine serum (USDA IMPORT TESTED FBS; Cytiva HyClone) was used as the medium.

#### 4.6.2. Evaluation of the Anticancer Effect of CBP-CMCG Using Colorectal Cancer Cells

The cytotoxicity test was performed using the Premix WST-1 cell proliferation assay system (Takara Bio Inc., Tokyo, Japan). HT-29 cells were seeded into 96-well plates at a density of 1 × 10^4^ cells/mL per well and incubated for 24 h at 37 °C in a 5% CO_2_ incubator. HT-29 cells used for the WST-1 assay were subcultured once prior to seeding. After incubation, the medium was completely removed, and 1.0 mg of CBP-CMCG with a CBP concentration of 1.0 mg per CMCG swollen in 110 µL of DMEM containing 10% FBS was added to each well. The positive control was 110 µL of DMEM containing 10% FBS and 0.5 mg of CBP, and the negative control was 110 µL of DMEM containing 10% FBS. For comparison, CMCG swollen in 110 µL of DMEM containing 10% FBS was used. Cells were incubated at 37 °C under 5% CO_2_ for 0, 1, 4, 6, 8, and 24 h. After each incubation period, 10 µL of WST-1 reagent was added to each well and further incubated for 2 h. The absorbance was measured at 440 nm with a reference wavelength of 660 nm using a SpectraMax Mini multimode microplate reader (Molecular Devices, LLC., San Jose, CA, USA). Cell viability (%) was calculated using Equation (3) and expressed as the mean of three independent experiments (*n* = 3), with standard deviations shown as error bars.

### 4.7. Microscopic Observation and Imaging of WI-38 and HT-29 Cells

Microscopic observations of WI-38 and HT-29 cells were performed using a ZEISS Primovert inverted microscope equipped with an Axiocam 208 color camera (Carl Zeiss AG, Oberkochen, Germany). The cells were examined unstained under phase-contrast optics. Initially, 4× and 10× objective lenses were used to assess the overall condition of the cells in the culture dishes, followed by detailed morphological observations using a 20× objective lens. Fields of view with minimal cell overlap, which allowed clear identification of both viable and dead cells, were selected for analysis. Representative images were obtained from the fields that best reflected the characteristic features of each sample.

## Data Availability

The data presented in this study, supporting the results, are available in the main text. Additional data are available upon request from the corresponding authors.

## Abbreviations

The following abbreviations are used in this manuscript:

AGU	Anhydroglucose Unit
CBP	Carboplatin
CBP-CMCG	Carboplatin-loaded Carboxymethyl Cellulose Hydrogel
CMC	Carboxymethyl Cellulose
CMCG	Carboxymethyl Cellulose Hydrogel
DMEM	Dulbecco’s Modified Eagle Medium
DS	Degree of Substitution
ECH	Epichlorohydrin
FBS	Fetal Bovine Serum
FTIR	Fourier Transform Infrared Spectroscopy
HT-29	Human Colorectal Adenocarcinoma Cell Line
JIS	Japanese Industrial Standard
MAS	Magic Angle Spinning
MEM	Minimum Essential Medium
NMR	Nuclear Magnetic Resonance
PBS	Phosphate-Buffered Saline
PS	Penicillin–Streptomycin
SEM	Scanning Electron Microscopy
WI-38	Human Normal Lung Fibroblast Cell Line
WST-1	Water-Soluble Tetrazolium-1 Cell Proliferation Assay
